# Determinants of Maternal Emotion Socialization: Based on Belsky’s Process of Parenting Model

**DOI:** 10.3389/fpsyg.2020.02044

**Published:** 2020-09-09

**Authors:** Jing Bao, Michiyo Kato

**Affiliations:** Department of Clinical Psychology, Graduate School of Education, Tohoku University, Sendai, Japan

**Keywords:** emotion socialization, emotion regulation, depression, anxiety, parenting alliance

## Abstract

The purpose of the current study was to investigate how the potential multifactors influence mothers’ emotion socialization. This study involved 300 Japanese-speaking married mothers with 2–5-year-old children, who answered a series of measures of emotion socialization (coaching, dismissing, dysfunction, and non-involvement), emotion regulation strategy (reappraisal and expressive suppression), psychopathology (anxiety and depression), and perceived parenting alliance with their partners. (a) Hierarchical multiple regression analyses demonstrated different effects between maternal anxiety and depression, such that higher levels of depression were associated with less coaching and higher levels of anxiety were associated with more dismissing and dysfunction. (b) Moreover, maternal emotion regulation was significant even when controlling for psychopathology, in which reappraisal had significant positive association with coaching and, conversely, expressive suppression had significant negative association with coaching and positive associations with non-involvement, dismissing, and dysfunction. (c) Additionally, moderation analysis revealed that a greater use of reappraisal was associated with more coaching, and this relation was strongest in lower levels of parenting alliance. Similarly, a greater use of reappraisal was associated with less dysfunction only when parenting alliance was low. Reappraisal may be effective in promoting supportive emotion socialization and buffering the negative effect of lower parenting alliance on unsupportive emotion socialization. Based on Belsky’s process of parenting model, we incorporate maternal psychopathology, emotion regulation, and perceived parenting alliance into one model of influencing maternal emotion socialization and highlight the unique role of emotion regulation.

## Introduction

Eisenberg et al. (1998) proposed that the process including parental response to children’s emotion, parent–child discussion of emotion, and parental own emotion expressivity is theoretically called emotion socialization (ES). ES is identified as emotion area-specific parenting and is embedded within general parenting. Emerging evidence shows that parental ES shapes children’s emotion repertoire, such as emotion recognition, expression, and regulation (Breaux et al., 2018; Wang et al., 2019), which in turn further impacts children’s psychological adjustment and peer relationships (Katz et al., 2012; Jin et al., 2017). For example, helping children identify the cause of angry feelings and teaching children how to address anger-causing problems may provide children with skills they can use next time when playing with a peer, thus potentially buffering their aggression words or behaviors (Nelson and Boyer, 2018). The impact from ES to offspring begins early in children’s lives and continues throughout adolescence, and even in adulthood. Key indicators of supportive ES include awareness, acceptance, and coaching of children’s emotion, and unsupportive ES forms include parents low on these facets.

Considering the critical outcome of parental ES on children’s socioemotional development, researchers began to examine why parents show different ES. The extant literature that is relevant to factors influencing parental ES has focused on children’s anxiety (Hastings et al., 2018) or parental marital conflict (Wong et al., 2009). However, it still needs to be verified how various factors affect certain ES dimensions independently or interactively. Belsky’s (1984) model (with Taraban and Shaw, 2018, updated) provided a theoretical perspective in understanding this mechanism, in which general parenting is primarily affected by factors relating to parental characteristics and family–social context. Thus, the purpose of the current study was to examine whether parental individual differences and the interplay with family context factors contribute to the way they socialize emotion in their offspring.

### Emotion Regulation and ES

In terms of parental individual cognitive and emotional factors, emotion regulation should be addressed since emotion regulation capacity is critical for parents. This is owing to their role of parenthood, which seems to endow people with a mission to behave in a well-regulated and sensitive manner when facing their offspring, especially on occasions when children display emotions (Rutherford et al., 2015).

In an important contribution to the literature, Gross (1998a) proposed the process model of emotion regulation. According to this model, individual differences of emotion regulation may arise at five points in the emotion-generative process: situation selection, situation modification, attentional deployment, cognitive change, and response modulation. People select emotion regulation strategies in order to achieve the goals of emotion regulation (e.g., decrease negative emotion). Two strategies are well studied: reappraisal and expressive suppression. Reappraisal is referred to as an antecedent-focused strategy of cognitive change, as emotional situations can be managed by modifying the emotional stimuli before the emotional response, whereas expressive suppression is a form of response modulation, in which people inhibit ongoing emotion expressive behavior (Gross, 1998b). Longer-term patterns of regulation strategy use reflect individual differences and are associated with cognitive (e.g., expressive suppression leads to low memory performance and reappraisal not), affective (e.g., expressive suppression leads to decrease in positive emotion experience, and reappraisal leads to decrease in negative emotion), and social (e.g., expressive suppression leads to less liking from social interaction partners, and reappraisal leads to greater liking from their peers) outcomes (Cutuli, 2014; Gross, 2014). Thus, reappraisal is identified as an adaptive strategy and expressive suppression as a maladaptive strategy.

Individual differences in emotion regulation impact parenting function. A systematic review yields partial support for this notion, indicating that poor control of emotions can lead to high risk of inappropriate parenting, whereas high capacity for emotion regulation is associated with sensitive, involved parenting (Crandall et al., 2015). Similarly, studies specific to ES reported a link between parental emotion dysregulation and ES. For example, researchers found positive relationships between adaptive emotion regulation strategy (reappraisal) and supportive ES behaviors (Shenaar-Golan et al., 2017) and negative relationships between maladaptive emotion regulation strategy (expressive suppression) and unsupportive ES behaviors (Rogers et al., 2016). Consistent with the outcomes, recent evidence indicated that observed maternal emotion regulation was negatively associated with unsupportive ES behaviors, whereas self-reported maternal emotion dysregulation was positively associated with unsupportive ES behaviors (Li et al., 2019).

However, most interestingly, scholars have postulated that the rules of “emotion regulation” are based on different cultures. Prior work has demonstrated that expressive suppression was associated with adverse psychological functioning for European Americans, but not for Chinese participants (Soto et al., 2011) or Asian Americans (Butler et al., 2007). In Western culture, the expressive suppression of emotion is equated with negative characteristics, such as passive personality, introversion, and low social competence, however, in Japanese culture, anger expressive suppression and other forms of expression control may be deemed as good social manners. This is perhaps because the value of interdependence and avoidance of conflicts with others took root in Eastern culture.

Thus, based on these studies, we hypothesized that mothers who score high on reappraisal tend to show more supportive ES to their children, and we did not put forth a hypothesis concerning expressive suppression due to the discrepancy discussed above.

### Psychopathology and ES

Well-documented literature has evidenced that parental psychopathology is a sensitive predictor of parenting and correlate to their own emotion regulation capacity. Researchers have found that emotion dysregulation is central to several clinical disorders and the onset of psychopathology (Gross and Muñoz, 1995; Shahar et al., 2017). Specifically, deficit of cognitive control and negative cognitive bias decrease the use of adaptive emotion regulation strategies, and this process exacerbates and sustains the negative mood that typifies depressive episodes (Le Moult and Gotlib, 2019). In these cases, to some degree, symptoms could share commonalities in the cognitive process with emotion regulation strategies. Thus, examining the mechanisms of emotion regulation accounting for certain ES dimensions is needed to control the impact of psychopathic symptoms.

A vast majority of research has focused on associations of maternal psychopathology and impaired parenting or sensitive parenting (Harder et al., 2017; Booth et al., 2018). Among those symptoms, maternal depression is most studied, since major depression disorder is particularly prevalent in mothers (Kessler, 2003). One study supports this relation by showing that maternal depression is negatively associated with their own sensitive response toward their children’s emotion expression (Behrendt et al., 2019). Another study added anxiety in addition to depression and found that both symptoms correlate with lower emotional awareness of their children (Moreira et al., 2019). Indeed, mothers’ low level of anxiety could be a significant predictor of a greater duration of maternal positive parenting behaviors (social positive engagement, offer of an object, and involvement in play) to their infants (Crugnola et al., 2016). Interestingly, one study highlighted the role of anxiety rather than depression, such that mothers with anxiety are observed to exhibit greater use of non-supportive reactions or not respond to children’s negative affect, however, these relations were not found in depression (Breaux et al., 2016). Therefore, the question of whether different impacts from anxiety and depression on ES dimensions exist needs more empirical evidence. Although anxiety was not included in the former model (Belsky, 1984; Taraban and Shaw, 2018), based on the above discussions, we involve anxiety into the current study and assume that anxiety and depression will be positively correlated with unsupportive maternal ES and negatively correlated with supportive maternal ES.

### Parenting Alliance and ES

In the social–family domain, the parenting alliance (also called coparenting), supportive or undermining, is at the center of the family system and many family interactions that impacts parenting (Feinberg, 2003). Parenting alliance reflects the quality of marital relationships and is concerned with parenthood. High-quality parenting alliance is characterized by a parent showing high respect for the other parent, approval of the other parent’s involvement with the child, and a desire for communication with the other parent (Weissman and Cohen, 1985).

We incorporate parenting alliance into our model for the following reasons. Firstly, maternal ES may be affected by family emotional climate, and this climate is highly correlated with the involvement of both fathers and mothers and their interactions. Secondly, one recent study reported that high-quality parenting alliance was associated with positive parenting (Becher et al., 2019). Nevertheless, impaired parenting alliance is predominant in families referred for parental psychopathology (e.g., maternal postpartum depression) and may lead to negative parenting. For instance, mothers are more likely to hinder fathers’ involvement in parenting when they have poorer psychological functioning (Schoppe-Sullivan et al., 2015). Thus, we hypothesized that high parenting alliance will be positively related to supportive ES and negatively related to unsupportive ES.

In sum, understanding how emotion regulation, psychopathology (depression and anxiety), and parenting alliance shape ES in mothers’ caregiving role (i.e., in parent–child interactions) would be particularly noteworthy. By exploring these factors, we can inform conceptual models and help identify targets for parent-oriented family prevention/intervention programs.

## Present Study

The present study was designed to examine how parental emotion regulation, anxiety, depression, and perceived parenting alliance affect ES by using a series of parental self-report scales. To summarize, we hypothesized effects of reappraisal, anxiety, depression, and parenting alliance on supportive ES and unsupportive ES. However, we did not predict the role of expressive suppression considering the potential influence from culture values since the participants are Japanese mothers in an Asian context. Besides, Taraban and Shaw’s (2018) model postulates the possibility of interactive contributions among family and individual facets. Thus, it is possible that parenting alliance may be one of the conditions that shapes individual psychological resource to ES. Regarding mothers in different levels of parenting alliance, hypotheses were of an exploratory nature.

## Materials and Methods

### Participants

Participants were recruited from the platform of the Cross Marketing Group Inc. by conducting an online survey across Japan. In total, the initial sample consisted of 300 Japanese-speaking mothers who had 2–5-year-old children and were all married. The answering time was computed by the *Fisher z*-transformation, and outliers were then removed at two standard deviations above the mean. The final sample was available for 286 mothers in the later analysis. Participants in the analyses ranged in age from 21 to 48 years old, and the mean age of mothers was 35.41 years (*SD* = 4.92, range = 21–48). In the current sample, of those mothers, 51.0% were housewives, 17.5% had full-time jobs, and 31.5% had part-time jobs or other types of work. In this sample, children of participants were, on average, 3.56 years old (*SD* = 1.12). Based on the data from the Ministry of Health, Labor and Welfare of Japan (2019), 54% of mothers who had infants stayed at home for caregiving, and this ratio decreased to 27% in mothers with 5-year-old children. Accordingly, our sample is normative in mothers with preschool-aged children. To clarify mothers’ perceived parenting alliance, they were asked whether they lived with their partners. Consequently, 99.0% (*N* = 283) of the participants lived with their partners, and only 1.0% (*N* = 3) of them did not. Of those children in our sample, 52.4% (*N* = 150) were girls, and 47.6% (*N* = 136) were boys. Only-child families accounted for 32.9%, and families with more than two children accounted for 67.1% of the sample.

### Measures

#### Emotion Socialization

Mothers completed the 24-item Japanese version of the Parental Meta-Emotion Philosophy about Anger Questionnaire (PMEPA-J; Bao and Kato, 2020; translated from Yeh, 2002) using a 7-point Likert scale (1 = “strongly disagree,” 7 = “strongly agree”). The PMEPA-J provides four subscales of ES styles. (a) coaching is characterized by parents’ validation of children’s emotion and tendency to find out what causes emotion, to soothe the child, to discuss the situations that elicited the emotions, and to provide the child with rules and strategies for coping with these situations (e.g., “First, I try to determine why the child becomes angry and then deal with it”); (b) dismissing (e.g., “I do not allow my child to express anger”) is characterized by parental invalidation or criticism of children’s emotions; (c) dysfunction is characterized by parental confusion toward children’s emotions and lack of self-control (e.g., “My child’s anger often makes my head explode”); and (d) non-involvement is characterized by a lack of regard for children’s emotions or a lack of any attempt to understand the causes of children’s emotions or to intervene (e.g., “There is no need to take the child’s anger seriously”). Coaching is identified as supportive ES; dismissing, dysfunction, and non-involvement are identified as unsupportive ES.

#### Emotion Regulation

Mothers completed the Japanese version of the Emotion Regulation Questionnaire (ERQ-J; Yoshizu et al., 2013; translated from Gross and John, 2003) using a 7-point Likert scale. The ERQ-J assesses individual differences in the usage of habitual, dispositional cognitive reappraisal and expressive suppression. The six-item reappraisal subscale is defined as changing the way one thinks about potentially emotion-eliciting events (e.g., “I control my emotions by changing the way I think about the situation I’m in”), and the four-item expressive suppression subscale is defined as changing the way one behaviorally responds to emotion-eliciting events (e.g., “I control my emotions by not expressing them”).

#### Depression

Maternal depression was assessed by the Japanese version of the Center for Epidemiologic Studies Depression Scale (CES-D) (Shima et al., 1985), which is designed to measure depressive symptomatology in the general population. Chiba Test Center Inc. (a corporation located in Tokyo, Japan) issued the license of this measure in 25 January 2019, for all 300 participants. Participants rated 20 items using a 4-point Likert scale (0–3 points) about the frequency of the occurrence of feelings during the past week: 0 = “rarely or none of the time,” 1 = “1 or 2 days,” 2 = “3 or 4 days,” and 3 = “more than 5 days.” Four items (4, 8, 12, and 16) are worded in a positive direction to reduce a tendency toward response bias; these items are reverse-coded. A higher score reflects greater symptoms of depression. A CES-D score greater than 16 is typically employed as a cutoff for clinical depression and usually warrants a referral for a more thorough diagnostic evaluation.

#### Anxiety

Mothers answered the trait anxiety subscale of the Japanese version of the State–Trait Anxiety Inventory (STAI; Shimizu and Imaei, 1981; translated from Spielberger et al., 1970). It consists of 20 items that are scored on four levels of anxiety intensity, from 1 = “not at all” to 4 = “very much,” and the summed scores range between 20 and 80. Higher scores indicate a greater severity of anxiety. The cutoff point is 46 according to Fisher and Durham (1999).

#### Parenting Alliance

Mothers completed the Japanese version of the Parenting Alliance Measure (PAM; Sato, 2008; translated from Abidin and Konold, 1999), which is used to assess the degree of commitment and cooperation between a mother and a father in child rearing. Psychological Assessment Resources, Inc. (a Florida corporation, with its principal offices located at 16204 North Florida Avenue, Lutz, Florida 33549) issued a license of this measure on 14 January 2019, for 300 participants. The PAM is a self-report instrument with 20 items measured on a 5-point Likert scale ranging from 1 = “strongly disagree” to 5 = “strongly agree,” with higher scores indicating a stronger and more positive alliance.

#### Demographics

Participants reported demographic information of themselves and their children; this information included mothers’ age and employment form, whether or not they were living with their partner, number of children in their families, and age and gender of the corresponding child who was 2–5 years old.

## Data Analytic Plan

IBM SPSS Statistical software version 22 was used to perform statistical analyses. Correlations among the variables were assessed using Pearson correlation coefficients, and Cronbach’s alphas of all scales were calculated for reliability. One-way analysis of variance (ANOVA) was performed to compare ES by mothers’ employment form (housewives, formal work, part-time job), and no group differences were found. Our first goal was to evaluate the unique effects of emotion regulation and psychopathology on different ES dimensions. The hierarchical multiple regression was conducted, and we included demographic variables (maternal age, child age, and the total number of children) as control variables. Our second goal was to evaluate whether the effects of emotion regulation differed depending on the levels of perceived parenting alliance from their partners. Thus, moderation analyses were performed using PROCESS macro (model 1; Hayes, 2013) with 5,000 bootstrap samples to test the interaction of parenting alliance and emotion regulation when predicting ES.

## Results

### Preliminary Analyses

Descriptive statistics of different variables are presented in Table 1. Reliability of all scales reached an adequate level with α = 0.70–0.95. Participants who reported clinical depression accounted for 31.5% (*N* = 90) of the sample, and those who reported clinical anxiety accounted for 52.4% (*N* = 150). Pearson correlation analyses indicated that mothers’ coaching was positively correlated with perceived parenting alliance (*r* = 0.26, *p* < 0.001) and cognitive reappraisal (*r* = 0.44, *p* < 0.001) and negatively correlated with depression (*r* = −0.28, *p* < 0.001) and anxiety(*r* = −0.24, *p* < 0.001). Conversely, mothers’ non-involvement, dismissing, and dysfunction were uncorrelated with parenting alliance and positively correlated with expressive suppression (*r* = 0.13–0.22), depression (*r* = 0.18–0.33), and anxiety (*r* = 0.18–0.37).

**TABLE 1:** Correlations and reliability for study variables.

		1	2	3	4	5	6	7	8	9	*α*	*M*	*SD*
1	EC	–									0.75	28.00	4.76
2	NON	−0.27***	–								0.77	16.90	4.92
3	DIS	−0.30***	0.55***	–							0.70	11.13	3.69
4	DF	−0.26***	0.37***	0.50***	–						0.86	9.40	4.10
5	Reappraisal	0.44***	0.01	–0.07	–0.12	–					0.83	24.49	5.13
6	Suppression	–0.02	0.22***	0.21***	0.13*	0.44***	–				0.77	13.26	4.04
7	PA	0.26***	–0.02	–0.09	–0.09	0.15*	0.04	–			0.95	68.46	15.11
8	Depression	−0.28***	0.18**	0.23***	0.33***	−0.25***	0.05	−0.34***	–		0.86	13.39	8.77
9	Anxiety	−0.24***	0.18**	0.29***	0.37***	−0.25***	0.06	−0.31***	0.76***	–	0.88	46.16	9.98

N = 286; EC, coaching; NON, non-involvement; DIS, dismissing; DF, dysfunction; PA, parenting alliance. ***p < 0.001, **p < 0.01, *p < 0.05.

### Hierarchical Multiple Regression Analyses

Hierarchical multiple regression analyses were conducted to answer the following question: Do emotion regulation predict ES, controlling for the impact of psychopathology? As displayed in Table 2, mothers’ age, children’s age, and the number of children in the family were entered at step 1 to control for

**TABLE 2:** Psychopathology and emotion regulation as predictors of ES.

Predictor		EC			NON			DIS			DF		
		*β*	*R*^2^	Δ *R*^2^	*β*	*R*^2^	Δ *R*^2^	*β*	*R*^2^	Δ *R*^2^	*β*	*R*^2^	Δ *R*^2^
Block 1			0.00			0.01			0.01			0.02	
	Maternal age	–0.05			0.01			0.03			0.07		
	Child’s age	0.01			–0.10			–0.01			–0.02		
	Number of children	0.00			–0.04			–0.07			−0.12^†^		
Block 2			0.09	0.08		0.05	0.04		0.09	0.08		0.16	0.14
	Maternal age	–0.08			0.03			0.05			0.11^†^		
	Child’s age	0.02			−0.11^†^			–0.02			–0.04		
	Number of children	–0.02			–0.02			–0.03			–0.07		
	Depression	−0.23**			0.12			0.02			0.15^†^		
	Anxiety	–0.07			0.10			0.28**			0.26**		
Block 3			0.27	0.19		0.09	0.05		0.14	0.05		0.18	0.02
	Maternal age	–0.07			0.02			0.04			0.10^†^		
	Child’s age	0.02			–0.10			–0.01			–0.04		
	Number of children	–0.03			–0.02			–0.03			–0.07		
	Depression	−0.16*			0.11			0.00			0.13		
	Anxiety	0.01			0.08			0.25**			0.24**		
	Reappraisal	0.50***			–0.04			−0.12^†^			–0.09		
	Suppression	−0.23***			0.23***			0.25***			0.15*		

EC, coaching; NON, non-involvement; DIS, dismissing; DF, dysfunction. ***p < 0.001, **p < 0.01, *p < 0.05, ^†^p < 0.10.

demographics. Maternal depression (CES-D) and anxiety (STAI-Trait) were entered at step 2, and then reappraisal and expressive suppression (ERQ-J) were entered at step 3. With reference to Table 2, at step 1, demographic variables were not significant predictors of maternal ES. However, maternal depression made significant contributions to the coaching regression model at step 2, with higher depression predicting lower coaching (*β* = −0.23, *p* < 0.01). On the other hand, higher anxiety predicted greater dismissing (*β* = 0.28, *p* < 0.01) and greater dysfunction (*β* = 0.26, *p* < 0.01). At step 3, after controlling for mothers’ mental health, maternal reappraisal served as a significant positive predictor of coaching (*β* = 0.50, *p* < 0.001), whereas expressive suppression was a negative predictor of coaching (*β* = −0.23, *p* < 0.001) and a positive predictor of non-involvement (*β* = 0.23, *p* < 0.001), dismissing (*β* = 0.25, *p* < 0.001), and dysfunction (*β* = 0.15, *p* < 0.05).

### Moderation Models

The set of moderation analyses were aimed to answer the following question: Does parenting alliance work as a moderator between the relation of emotion regulation and ES? The following effects were significant: the moderation effect of parenting alliance on the relationship between reappraisal and coaching or on that between reappraisal and dysfunction. The interaction term *parenting alliance* × *reappraisal* was significant for coaching (*b* = −0.01, *p* = 0.024 < 0.05) and dysfunction (*b* = 0.01, *p* = 0.021 < 0.05) and was non-significant for dismissing and non-involvement (Table 3). In terms of expressive suppression, the interaction term was not significant in either ES dimension.

**TABLE 3:** Parenting alliance and cognitive reappraisal predicting emotion socialization.

	EC		NON		DIS		DF	
	*b*	*SE*	*b*	*SE*	*B*	*SE*	*b*	*SE*
Reappraisal	0.37***	0.05	0.01	0.06	–0.04	0.04	–0.07	0.05
PA	0.06***	0.02	–0.01	0.02	–0.02	0.01	–0.02	0.02
Reappraisal × PA	−0.01*	0.00	0.00	0.00	0.00	0.00	0.01*	0.00

EC, coaching; NON, non-involvement; DIS, dismissing; DF, dysfunction; PA, parenting alliance. ***p < 0.001, **p < 0.01, *p < 0.05.

**FIGURE 1:**
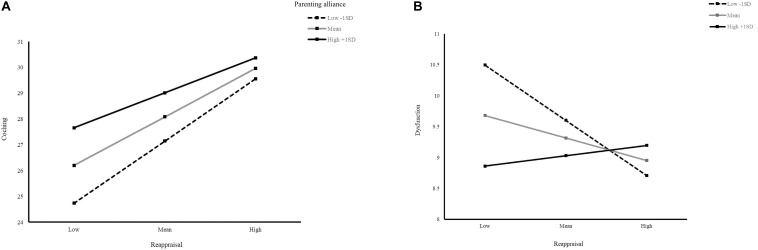
**(A)** Moderation model of parenting alliance on reappraisal and emotinal-coaching. Slopes of parenting alliance at the mean, as well as ± 1 SD from the mean. Reapprasial is on the *x*-axis and emotion-coaching is on the *y*-axis. **(B)** Moderation model of parenting alliance on reappraisal and emotinal-dysfunction. Slopes of parenting alliance at the mean, as well as ± 1 SD from the mean. Reapprasial is on the *x*-axis and emotion-dysfunction is on the *y*-axis.

Then, simple slopes were probed for significant interactions at one standard deviation above and below mean levels of parenting alliance. For coaching, the simple slopes (see Figure 1A) were as follows for different levels of parenting alliance: *b* = 0.47 [*t*(282) = 7.49, *p* < 0.001] at low levels of parenting alliance (-1 *SD*); *b* = 0.37 [*t*(282) = 7.52, *p* < 0.001] at moderate levels of parenting alliance (mean); and *b* = 0.26 [*t*(282) = 3.74, *p* < 0.001] at high levels of parenting alliance (1 *SD*). These results revealed that mothers who reported poor reappraisal were more likely to have high coaching in low levels of parenting alliance. For dysfunction, the simple slope (see Figure 1B) for low levels of parenting alliance (-1 *SD*) was *b* = −0.17 [*t*(282) = −2.85, *p* < 0.01] and significant. The non-significant simple slope for moderate levels of parenting alliance was *b* = −0.07 [*t*(282) = −1.49, *p* = 0.13], and that for high levels of parenting alliance (1 *SD*) was *b* = 0.03 [*t*(282) = 0.47, *p* = 0.64]. These results revealed that reappraisal negatively predicted dysfunction when lower levels of parenting alliance were reported. This association was not found in mothers who reported moderate and high levels of parenting alliance.

## Discussion

**FIGURE 2:**
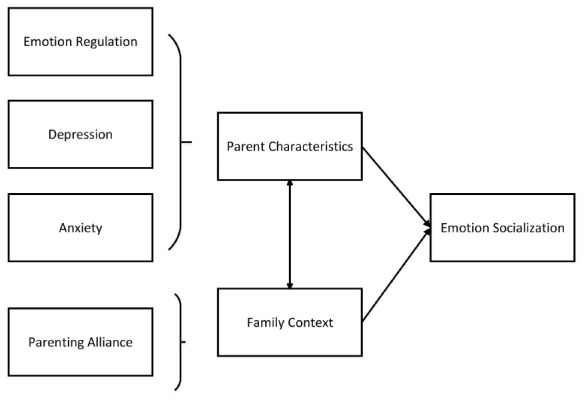
Theoretical model of emotion socialization.

The strength of this study in psychology is that it extends the influential factor model of parenting formulated by Belsky (1984) and Taraban and Shaw (2018), exclusively on emotion-related parenting. We added new factors of emotion regulation, anxiety, and most uniquely, parenting alliance to the current conceptual model (Figure 2). To our knowledge, no studies thus far incorporated the crucial role that emotion regulation may play in ES by considering the psychopathology and family contextual factors simultaneously. This study addressed this gap by including the role of maternal emotion regulation on their own ES and interactive role with other factors.

### Reappraisal and Expressive Suppression Link With ES

With respect to emotion regulation, as expected, the present study indicated that mothers who relied more on reappraisal strategies in daily life tended to adopt more supportive ES; in contrast, mothers who relied more on expressive suppression tended to adopt less supportive ES (coaching) and more unsupportive ES (dismissing, non-involvement, and dysfunction). These results are still significant when controlling for shared variances of depression and anxiety, and consistent with prior studies. For example, compared to expressive suppression, reappraisal results in less negative general parenting behaviors (overreactive and lax discipline; Lorber, 2012) and specifically less negative physiological response of infant crying (Riem and Karreman, 2019). The correlations may be interpreted by the view that mothers who are readily able to regulate their own emotions are likely to experience healthier patterns of affect and well-being and then express less negative affect and more patience when the child displays negative emotions. By contrast, the tendency of expressive suppression use in mothers’ own emotion displays may thus impact mothers’ ES goals and then lead to their endorsement of beliefs stressing that parents should not get involved in children’s emotion displays and should stop children from expressing emotions. Considering culture values, we did not expect any directions from expressive suppression to ES. Consequently, in the current study, we have not found different effects of suppression compared with Western data.

In sum, the results of our study underscore the importance of emotion regulation in mother’s daily life. Parental ES is demonstrated to be one of the consequences of implementing different emotion regulation strategies. Of interest, the effect of maternal emotion regulation on ES may explain why close partners (parent–child dyads and marital dyads) exhibit a pattern of emotional similarity across time (Mercado et al., 2019) and provide basics for the mechanism of the intergenerational transmission of emotion regulation skills from parents to their offspring through ES (Morelen et al., 2016).

### Reappraisal as a Protective Factor Against Poor Parenting Alliance’s Detrimental Effect on ES

Our findings are the first to consider whether parental emotion regulation and parenting alliance interact to predict ES. Mothers with stronger parenting alliance have high-quality, cooperative relationships with their partners with respect to childcare, which reflect healthy parenting environment and may mitigate parenting stress (Choi and Becher, 2019). Therefore, we expected main effects of parenting alliance on the four ES dimensions, and consequently, the main effect was only found in coaching. The result that stronger parenting alliance is associated with more coaching suggests that supportive ES is most likely to develop when caregivers perceive secure coparentive bonds with their partners. From the perspective of family systems theory, this result for parenting alliance strengthens the influence of the father–mother dyad systems on mother–child systems.

Moreover, intriguingly, parenting alliance significantly moderated the relationship between reappraisal and coaching or between reappraisal and dysfunction. The moderation models suggest that when mothers are exposed to low parenting alliance (less father involvement or more interparental conflicts), higher levels of reappraisal are more likely to promote coaching and mitigate dysfunction. However, the moderation effects are not significant with respect to moderate and high levels of parenting alliance in dysfunction. These findings may suggest that mothers’ individual tendencies in emotion regulation contribute more to unfavorable positions, such as a lack of parenting cooperation, support, or resources from partners. In other words, reappraisal seems to be a protective factor against poor parenting alliance’s detrimental effect on ES. This result is particularly important for Japanese mothers, because they spent more time in household activities (7.34 h) and childcare (3.45 h) than Japanese fathers (1.23 h and 0.49 h, respectively) on an average day (Ministry of Internal Affairs and Communications, 2016). Thus, Japanese mothers take a major role in caregiving and face challenges dealing with children’s emotional situations far more than fathers do. Our results provide a possible solution to help mothers in low parenting alliance, to avoid adopting unsupportive ES styles by improving their own emotion regulation skills.

### Role of Psychopathology on ES

We examined symptoms of depression and anxiety after controlling for demographic variables. As expected, higher depression symptoms were significantly related to lower coaching but were unrelated to any unsupportive ES styles. This result is consistent with prior studies, in which parental depression could predict withdrawal parenting, characterized by high disengagement and low responsiveness (Vreeland et al., 2019). Additionally, mothers who are depressed may lack certain parenting attributes, such as warmth (Mitchell et al., 2019), sensitivity, and the ability to provide structure in children’s environments in ways that promote the acquisition of the skills necessary for successful emotion regulation (Hoffman et al., 2006). And these abilities are core components of coaching, as coaching parents discuss and label emotion and teach the child about the display rules of emotion.

In contrast, higher anxiety symptoms were significantly associated with more dismissing and dysfunction. One possible explanation is that anxiety is highly characterized by sensory overresponsivity (Amos et al., 2019), which refers to the subjective experience of sensory overload, such as noise or light. This feature suggests a possibility for higher-anxiety parents to show overresponse to children’s anger expressions, thus resulting in denying the children’s display of anger (dismissing) or becoming impulsive and reckless about the emotional situations (dysfunction).

We concluded that depression hinders coaching and that anxiety facilitates dismissing and dysfunction. Additionally, we found no significant prediction by depression or anxiety on non-involvement. The effects of non-involvement were different from that of Lovejoy et al.’s (2000) meta-analysis, in which a moderate effect of maternal depression on disengaged parenting (similar with non-involvement) was found.

Our results of psychopathology suggest that parental symptoms impaired emotion-related parenting function. This could be in line with a systemic review, in which mothers with anxiety or depression have difficulty identifying infant positive emotions but are more accurate at recognizing infant negative emotions and are less easily distracted from infant emotions (Webb and Ayers, 2015). Based on this work, parents with symptoms tend to pay excessive attention to children’s negative emotion and lack the skills necessary to draw wisdom from emotion-related incidents, which in turn are indicative of less supportive ES and more unsupportive ES.

## Implications, Limitations, and Future Work

The current study is not without limitations. First, given the methodological limitation stemming from using mothers’ reports of their own ES as well as other predictive factors, we refrain from drawing strong conclusions regarding casual relations based on the current data. Specifically, in terms of psychopathology, self-report scales lack an assessment of the duration of symptoms (Hoffman et al., 2006). Hence, in the current study, our findings were not very sensitive to reflect the impact from chronic emotional disorders. Second, with respect to ES, the scale PMEPA-J used in our study focuses on parental response to children’s anger since children’s anger was found to be strongly related to externalizing (aggression) problems (Barnes et al., 2017) or internalizing problems (Sanders et al., 2015). We also suggest further replication across different types of children’s emotions. Therefore, the conclusions in the current study could not be simply interpreted when discussing parental ES to children’s other types of emotions (e.g., happiness, sadness, and fear). Besides, this study assessed general emotion regulation in mothers’ daily life and did not assess emotion regulation within the parenting context, which may be a more proximal predictor of ES.

Moreover, future research would also benefit from examining influences from ES to children within mother–father–child triadic interaction, as coparent ES (Thomassin et al., 2017) or fathers’ observed ES (Gerhardt et al., 2020) may be critical factors in children’s emotion regulation, mental health, and emotion expression. Finally, future longitudinal studies could highlight the individual and familial mechanisms that contribute to this topic.

Despite these limitations, our findings provide important new insights into understanding the mechanisms on the relation of parental internal factors, family context factors, and parental own ES. And this study raises important policy and practice implications. It is possible that emotion dysregulation can be important to identify mothers who are likely to have trouble in ES, and emotion regulation can be an important intervention entry point that helps individuals build a healthy ES style. We anticipate an incorporation of these skills into daily parenting practices and future government–social service systems that integrate parental education and support, maltreatment prevention, and early child development by focusing on factors that influence ES indirectly (i.e., how to improve emotion regulation) or directly (how to coach children’s emotions).

## Conclusion

In this study, we found that reappraisal was related to more coaching, whereas expressive suppression was related to less coaching and more dismissing, non-involvement, and dysfunction. And these linkages were also significant after controlling for parental anxiety and depression. In addition, parental anxiety was linked with more dismissing and dysfunction, and parental depression was linked with more coaching. Finally, parenting alliance moderated the relation between reappraisal and coaching or between reappraisal and dysfunction. These results shed new light on theories of parental ES and offer empirical evidence in non-Western cultures.

## Data Availability Statement

The datasets generated for this study are available on request to the corresponding author.

## Ethics Statement

The studies involving human participants were reviewed and approved by the Ethics Committee of Graduate School of Education, Tohoku University. Written informed consent for participation was not required for this study in accordance with the national legislation and the institutional requirements.

## Author Contributions

JB conceived of the presented idea, performed the analysis, and drafted the manuscript. MK was involved in planning and supervised the work, the design, and implementation of the research. Both authors contributed to the article and approved the submitted version.

## Conflict of Interest

The authors declare that the research was conducted in the absence of any commercial or financial relationships that could be construed as a potential conflict of interest.

## References

[B1] Abidin R. R. Konold T. (1999). *Parenting Alliance Measure - Professional manual.* Odessa, FL: Psychological Assessment Resources.

[B2] Amos G. A. Byrne G. Chouinard P. A. Godber T. (2019). Autism traits, sensory over-responsivity, anxiety, and stress: a test of explanatory models. *J. Autism Dev. Disord.* 49 98–112. 10.1007/s10803-018-3695-6 30043351

[B3] Bao J. Kato M. (2020). The Japanese version of the parental meta-emotion philosophy about anger questionnaire: a psychometric evaluation. *Jpn. J. Psychol.* 91 165–172. 10.4992/jjpsy.91.19208

[B4] Barnes S. E. Howell K. H. Thurston I. B. Cohen R. (2017). Children’s attitudes toward aggression: associations with depression, aggression, and perceived maternal/peer responses to anger. *J. Child Fam. Stud.* 26 748–758. 10.1007/s10826-016-0612-5

[B5] Becher E. H. Kim H. Cronin S. E. Deenanath V. McGuire J. K. McCann E. M. (2019). Positive parenting and parental conflict: contributions to resilient coparenting during divorce. *Fam. Relat.* 68 150–164. 10.1111/fare.12349

[B6] Behrendt H. F. Scharke W. Herpertz-Dahlmann B. Konrad K. Firk C. (2019). Like mother, like child? Maternal determinants of children’s early social-emotional development. *Infant Men. Health J.* 40 234–247. 10.1002/imhj.21765 30731022

[B7] Belsky J. (1984). The determinants of parenting: a process model. *Child Dev.* 55 83–96. 10.2307/1129836 6705636

[B8] Booth A. T. Macdonald J. A. Youssef G. J. (2018). Contextual stress and maternal sensitivity: a meta-analytic review of stress associations with the maternal behavior Q-Sort in observational studies. *Dev. Rev.* 48 145–177. 10.1016/j.dr.2018.02.002

[B9] Breaux R. P. Harvey E. A. Lugo-Candelas C. I. (2016). The role of parent psychopathology in emotion socialization. *J. Abnorm. Child Psychol.* 44 731–743. 10.1007/s10802-015-0062-3 26267238 PMC4752927

[B10] Breaux R. P. McQuade J. D. Harvey E. A. Zakarian R. J. (2018). Longitudinal associations of parental emotion socialization and children’s emotion regulation: the moderating role of ADHD symptomatology. *J. Abnorm. Child Psychol.* 46 671–683. 10.1007/s10802-017-0327-0 28710531

[B11] Butler E. A. Lee T. L. Gross J. J. (2007). Emotion regulation and culture. *Emotion* 7 30–48. 10.1037/1528-3542.7.1.30 17352561

[B12] Choi J. K. Becher E. H. (2019). Supportive coparenting, parenting stress, harsh parenting, and child behavior problems in nonmarital families. *Fam. Process* 58 404–417. 10.1111/famp.12373 29924390

[B13] Crandall A. Deater-Deckard K. Riley A. W. (2015). Maternal emotion and cognitive control capacities and parenting: a conceptual framework. *Dev. Rev.* 36 105–126. 10.1016/j.dr.2015.01.004 26028796 PMC4445866

[B14] Crugnola C. R. Ierardi E. Ferro V. Gallucci M. Parodi C. Astengo M. (2016). Mother-infant emotion regulation at three months: the role of maternal anxiety, depression and parenting stress. *Psychopathology* 49 285–294. 10.1159/000446811 27410251

[B15] Cutuli D. (2014). Cognitive reappraisal and expressive suppression strategies role in the emotion regulation: an overview on their modulatory effects and neural correlates. *Front. Syst. Neurosci.* 8:175. 10.3389/fnsys.2014.00175 25285072 PMC4168764

[B16] Eisenberg N. Cumberland A. Spinrad T. L. (1998). Parental socialization of emotion. *Psychol. Inq.* 9 241–273. 10.1207/s15327965pli0904_1 16865170 PMC1513625

[B17] Feinberg M. E. (2003). The internal structure and ecological context of coparenting: a framework for research and intervention. *Parent. Sci. Pract.* 3 95–131. 10.1207/S15327922PAR0302_01 21980259 PMC3185375

[B18] Fisher P. L. Durham R. C. (1999). Recovery rates in generalized anxiety disorder following psychological therapy: an analysis of clinically significant change in the STAI-T across outcome studies since 1990. *Psychol. Med.* 29 1425–1434. 10.1017/s0033291799001336 10616949

[B19] Gerhardt M. Feng X. Wu Q. Hooper E. G. Ku S. Chan M. H. (2020). A naturalistic study of parental emotion socialization: unique contributions of fathers. *J. Fam. Psychol.* 34 204–214. 10.1037/fam0000602 31670561

[B20] Gross J. J. (1998a). The emerging field of emotion regulation: an integrative review. *Rev. Gen. Psychol.* 2 271–299. 10.1037/1089-2680.2.3.271

[B21] Gross J. J. (1998b). Antecedent- and response-focused emotion regulation: Divergent consequences for experience, expression, and physiology. *J. Pers. Soc. Psychol.* 74 224–237. 10.1037/0022-3514.74.1.224 9457784

[B22] Gross J. J. (2014). “Emotion regulation: conceptual and empirical foundations,” in *Handbook of Emotion Regulation*, 2nd Edn, ed. Gross J. J. (New York, NY: Guilford Press), 9–11.

[B23] Gross J. J. John O. P. (2003). Individual differences in two emotion regulation processes: implications for affect, relationships, and well-being. *J. Pers. Soc. Psychol.* 85 348–362. 10.1037/0022-3514.85.2.348 12916575

[B24] Gross J. J. Muñoz R. F. (1995). Emotion regulation and mental health. *Clin. Psychol. Sci. pract.* 2 151–164. 10.1111/j.1468-2850.1995.tb00036.x

[B25] Harder A. T. Knorth E. J. Kalverboer M. E. Tausendfreund T. Knot-Dickscheit J. (2017). Parental perspectives: risk and protective factors associated with parenting quality for parents of adolescents in secure residential care. *Child Fam. Soc. Work* 23 549–557. 10.1111/cfs.12404

[B26] Hastings P. D. Grady J. S. Barrieau L. E. (2018). Children’s anxious characteristics predict how their parents socialize emotions. *J. Abnorm. Child Psychol.* 47 1225–1238. 10.1007/s10802-018-0481-z 30367307

[B27] Hayes A. F. (2013). *Model Templates for PROCESS for SPSS and SAS.* New York, NY: Guilford Press.

[B28] Hoffman C. Crnic K. A. Baker J. K. (2006). Maternal depression and parenting: implications for children’s emergent emotion regulation and behavioral functioning. *Parent. Sci. Pract.* 6 271–295. 10.1207/s15327922par0604_1

[B29] Jin Z. Zhang X. Han Z. R. (2017). Parental emotion socialization and child psychological adjustment among Chinese urban families: mediation through child emotion regulation and moderation through dyadic collaboration. *Front. Psychol.* 8:2198. 10.3389/fpsyg.2017.02198 29326629 PMC5741672

[B30] Katz L. F. Maliken A. C. Stettler N. M. (2012). Parental meta-emotion philosophy: a review of research and theoretical framework. *Child Dev. Perspect.* 6 417–422. 10.1111/j.1750-8606.2012.00244.x

[B31] Kessler R. C. (2003). Epidemiology of women and depression. *J. Affect. Disord.* 74 5–13. 10.1016/s0165-0327(02)00426-3 12646294

[B32] Le Moult J. Gotlib I. H. (2019). Depression: a cognitive perspective. *Clin. Psychol. Rev.* 69 51–66. 10.1016/j.cpr.2018.06.008 29961601 PMC11884012

[B33] Li D. Li D. Wu N. Wang Z. (2019). Intergenerational transmission of emotion regulation through parents’ reactions to children’s negative emotions: tests of unique, actor, partner, and mediating effects. *Child. Youth Serv. Rev.* 101 113–122. 10.1016/j.childyouth.2019.03.038

[B34] Lorber M. F. (2012). The role of maternal emotion regulation in overreactive and lax discipline. *J. Fam. Psychol.* 26 642–647. 10.1037/a0029109 22888786 PMC4523636

[B35] Lovejoy M. C. Graczyk P. A. O’Hare E. Neuman G. (2000). Maternal depression and parenting behavior: a meta-analytic review. *Clin. Psychol. Rev.* 20 561–592. 10.1016/S0272-7358(98)00100-7 10860167

[B36] Mercado E. Kim J. Gonzales N. A. Fuligni A. J. (2019). Emotional coregulation in Mexican-origin parent–adolescent dyads: associations with adolescent mental health. *Journal of Youth and Adolescence.* 48 1116–1130. 10.1007/s10964-019-01002-5 30830533

[B37] Ministry of Health, Labor and Welfare of Japan (2019). *Basic Life Investigation of Citizens in 2018.* Tokyo: Ministry of Health, Labor and Welfare of Japan.

[B38] Ministry of Internal Affairs and Communications (2016). *Basic Life Investigation of Citizens in 2016.* Tokyo: Ministry of Internal Affairs and Communications.

[B39] Mitchell E. A. Nuttall A. K. Wittenborn A. (2019). Maternal depressive symptoms and warm responsiveness across the transition to parenthood. *J. Child Fam. Stud.* 28 1604–1612. 10.1007/s10826-019-01392-x

[B40] Moreira H. Fonseca A. Caiado B. Canavarro M. C. (2019). Work-family conflict and mindful parenting: the mediating role of parental psychopathology symptoms and parenting stress in a sample of Portuguese employed parents. *Front. Psychol.* 10:635. 10.3389/fpsyg.2019.00635 30967822 PMC6438855

[B41] Morelen D. Shaffer A. Suveg C. (2016). Maternal emotion regulation: links to meta-emotion philosophy and child emotion regulation. *J. Fam. Issues* 37 1891–1916. 10.1177/0192513x14546720

[B42] Nelson J. A. Boyer B. P. (2018). Maternal responses to negative emotions and child externalizing behavior: different relations for 5-, 6-, and 7-year-olds. *Soc. Dev.* 27 482–494. 10.1111/sode.12296

[B43] Riem M. M. Karreman A. (2019). Experimental manipulation of emotion regulation changes mothers’ physiological and facial expressive responses to infant crying. *Infant Behav. Dev.* 55 22–31. 10.1016/j.infbeh.2019.02.003 30826499

[B44] Rogers M. L. Halberstadt A. G. Castro V. L. MacCormack J. K. Garrett-Peters P. (2016). Maternal emotion socialization differentially predicts third-grade children’s emotion regulation and lability. *Emotion* 16 280–291. 10.1037/emo0000142 26641269

[B45] Rutherford H. J. Wallace N. S. Laurent H. K. Mayes L. C. (2015). Emotion regulation in parenthood. *Dev. Rev.* 36 1–14. 10.1016/j.dr.2014.12.008 26085709 PMC4465117

[B46] Sanders W. Zeman J. Poon J. Miller R. (2015). Child regulation of negative emotions and depressive symptoms: the moderating role of parental emotion socialization. *J. Child Fam. Stud.* 24 402–415. 10.1007/s10826-013-9850-y

[B47] Sato N. (2008). The relationship between parenting alliance and conditions of mutual support in parents of preschool children with special needs. *Chiba Acad. Nurs. Sci.* 14 46–53.

[B48] Schoppe-Sullivan S. J. Altenburger L. E. Lee M. A. Bower D. J. Kamp Dush C. M. (2015). Who are the gatekeepers? Predictors of maternal gatekeeping. *Parenting* 15 166–186. 10.1080/15295192.2015.1053321 27366115 PMC4922533

[B49] Shahar B. Bar-Kalifa E. Alon E. (2017). Emotion-focused therapy for social anxiety disorder: results from a multiple-baseline study. *J. Consult. Clin. Psychol.* 85 238–249. 10.1037/ccp0000166 28221059

[B50] Shenaar-Golan V. Wald N. Yatzkar U. (2017). Patterns of emotion regulation and emotion-related behaviors among parents of children with and without ADHD. *Psychiatry Res.* 258 494–500. 10.1016/j.psychres.2017.08.090 28890229

[B51] Shima S. Shikano T. Kitamura T. Asai M. (1985). New self-rating scales for depression. *Psychiatry* 27 717–723.

[B52] Shimizu H. Imaei K. (1981). Construction of a Japanese version of the state-trait anxiety inventory. *Jpn. J. Educ. Psychol.* 29 348–353. 10.5926/jjep1953.29.4_348

[B53] Soto J. A. Perez C. R. Kim Y. Lee E. A. Minnick M. R. (2011). Is expressive suppression always associated with poorer psychological functioning? A cross-cultural comparison between European Americans and Hong Kong Chinese. *Emotion* 11 1450–1455. 10.1037/a0023340 21707152

[B54] Spielberger C. D. Gorsuch R. L. Lushene R. E. (1970). *Manua1 for the State-Trait Anxiety Inventory(Self-Evaluation Questionnaire).* Palo Alto, CA: Consulting Psychologists Press.

[B55] Taraban L. Shaw D. S. (2018). Parenting in context: revisiting Belsky’s classic process of parenting model in early childhood. *Dev. Rev.* 48 55–81. 10.1016/j.dr.2018.03.006

[B56] Thomassin K. Suveg C. Davis M. Lavner J. A. Beach S. R. (2017). Coparental affect, children’s emotion dysregulation, and parent and child depressive symptoms. *Fam. Process* 56 126–140. 10.1111/famp.12184 26384583

[B57] Vreeland A. Gruhn M. A. Watson K. H. Bettis A. H. Compas B. E. Forehand R. (2019). Parenting in context: associations of parental depression and socioeconomic factors with parenting behaviors. *J. Child Fam. Stud.* 28 1124–1133. 10.1007/s10826-019-01338-3 PMC12829980 41583615

[B58] Wang M. Liang Y. Zhou N. Zou H. (2019). Chinese fathers’ emotion socialization profiles and adolescents’ emotion regulation. *Pers. Indiv. Differ.* 137 33–38. 10.1016/j.paid.2018.08.006

[B59] Webb R. Ayers S. (2015). Cognitive biases in processing infant emotion by women with depression, anxiety and post-traumatic stress disorder in pregnancy or after birth: a systematic review. *Cogn. Emot.* 29 1278–1294. 10.1080/02699931.2014.977849 25472032

[B60] Weissman S. H. Cohen R. S. (1985). The parenting alliance and adolescence. *Adoles. Psychiatry* 12 24–45. 4003682

[B61] Wong M. S. McElwain N. L. Halberstadt A. G. (2009). Parent, family, and child characteristics: associations with mother- and father-reported emotion socialization practices. *J. Fam. Psychol.* 23 452–463. 10.1037/a0015552 19685981

[B62] Yeh K. H. (2002). “Parental meta-emotion philosophy styles and measures,” in *Affect, Emotion and Culture: Anthropological and Psychological Studies in Taiwanese Society*, eds Hu T. L. Hsu M. Yeh K. H. (Taipei: Institute of Ethnology Academia Sinica), 268–297.

[B63] Yoshizu J. Sekiguchi R. Amemiya T. (2013). Development of a Japanese version of emotion regulation questionnaire. *Jpn. J. Res. Emot.* 20 56–62. 10.4092/jsre.20.56

