# Supplementary material for: Determinants of Maternal Emotion Socialization: Based on Belsky’s Process of Parenting Model
Source: Front Psychol. 2020 Sep 9;11:2044. doi: 10.3389/fpsyg.2020.02044 (PMC7509056; doi:10.3389/fpsyg.2020.02044)
Supplement: Supplementary file 1 [file Table_1.pdf]

Supplementary Material

Table 1 Confirmatory Factor Analysis of PMEPA-J

	Items(Japanese)	Items(English)	Factor Loading	*M*	*SD*
**F1:非関与 *α*＝.82，*ω*＝.87**		**Non-Involvement :**			
**15**	子どもが怒ったことを気にかける必要はない	There is no need to take the child's anger seriously.	.83	2.49	1.21
**11**	子どもが怒った理由など気にかける必要はないと思う	There is no need to worry about the cause of the child's anger.	.77	2.36	1.25
**10**	子どもは自分の怒りを自分で何とかすべきであり，私が何かしてあげられることはなにもない	The child should deal with anger by himself(herself), and I can do nothing.	.72	2.95	1.30
**13**	子どもが怒ったとき，親は干渉すべきではないと思う	When a child becomes angry, I think parents should not interfere with it.	.67	3.08	1.15
**4**	子どもが怒ったときは，実は何をしても別に意味がない。	When a child is angry, it is useless to do anything as parents.	.52	3.39	1.31
**20**	子どもは怒るのが当然なので，別に気にかける必要はないと思う	It is naturally that children become angry, there is no need to make a big deal.	.43	3.44	1.34
**F2:機能不全 *α*＝.89，*ω*＝.89**		**Dysfunction :**			
**7**	子どもが怒ると，私はパニックになって頭が爆発しそうになる	My child's angry often makes my head explode.	.90	2.66	1.48
**8**	子どもが怒ると，私はどうすればいいのかわからなくなる	When my child gets angry, I do not know what to do.	.83	2.83	1.47
**6**	子どもが怒ると，私は自分の感情をコントロールできなくなる	When my child gets angry, I always lose control of my own emotions.	.83	3.22	1.43
**17**	怒っている子どもと冷静に話し合うことは，私には難しい [a]	It is difficult for me to talk with an angry child. [a]		3.64	1.43
**2**	私は，子どもが怒ったときの私の対応に対して後悔ばかりしている [a]	I often regret what I did with my child when he(she) is angry. [a]		4.18	1.26

3	私は，子どもがなぜ怒るのかがさっぱりわからない [a]	I completely do not know why children become angry. [a]		3.09	1.19
**F3:軽視** *α*＝.76，*ω*＝.78		**Dismissing:**			
19	私は，子どもが怒りを表すことを許さない	I do not allow my child to express anger.	.79	2.23	1.29
18	子どもがいつも同じようなことで怒るなら，私は罰を与える	When my child becomes angry for repeated reasons, I would punish him(her).	.69	3.14	1.39
16	子どもの怒りは，いつも大した理由はなく，理屈に合わないことばかりだと思う	Children’s anger always has no particular reasons and make no sense.	.62	3.20	1.33
24	子どもが怒ったら，私はすぐに子どもを制止して怒るのをやめさせる	When my child becomes angry, I will soon let him(her)stop expressing anger.	.55	3.21	1.24
23	子どもの怒りは心身の成長のためによくないと思う [a]	Children’s anger is harmful to their development. [a]		2.77	1.25
14	たとえどんな理由があっても，子どもは周囲に八つ当たりすべきではない [a]	Children should not kick-the-cat on any account. [a]		4.09	1.47
**F4:コーチング** *α*＝.75，*ω*＝.80		**Coaching:**			
21	私は，子どもが怒った時に示す表情や反応について，気にかけるようにしている	I aware of the child’s facial expression and response when he(she)is angry.	.73	4.85	1.17
9	私は，まず子どもが怒った理由を理解してから対応するように気をつけている	First, I try to figure out why the child becomes angry and then deal with it.	.71	4.81	1.19
22	私は，感情を適切に表現する方法を子どもに教える	I teach my child how to express emotions appropriately.	.58	4.51	1.20
1	私は子どもの怒りの感情をとても大事なものと考えている	I think children’s angry is an important thing.	.58	4.84	1.20
12	子どもが怒りを示すことで，私はむしろ子どものことをよく理解できる	I can understand my child better by his(her)angry.	.48	4.32	1.07
5	子どもが怒ったとき，私は子どもをなだめて落ち着かせる	When my child becomes angry, I offer him(her) comfort.	.40	4.43	1.26
	Correlations of latent variables		F2	F3	F4
	F1		.62	.88	−.58
	F2			.66	−.39
	F3				−.54

Note [a] Deleted items.
PMEPA-J, Japanese version of the Parental Meta-Emotion Philosophy about Anger Questionnaire.

The Japanese version of the Parental Meta-Emotion Philosophy about Anger Questionnaire (PMEPA-J) yielded a 19- item, 4-factor structure with the following factors: Coaching, Non-Involvement, Dismissing, and Dysfunction. Items in English are translated from Japanese version.

For more information, please refer to :

Bao, J., and Kato, M. (2020). The Japanese version of the parental meta-emotion philosophy about anger questionnaire: a psychometric evaluation. The Japanese Journal of Psychology,91(3), 165-172. https://doi.org/10.4992/jjpsy.91.19208
